# Zoonotic *Centrocestus formosanus* (Digenea: Heterophyidae) infecting *Melanoides tuberculata* (Mollusca: Thiaridae) in Ecuador. Genetic characterization

**DOI:** 10.1016/j.ijppaw.2026.101228

**Published:** 2026-04-02

**Authors:** Carlos Bastidas-Caldes, Fernanda Hernández-Alomia, Juan Pablo Negrete, Manuel Calvopina

**Affiliations:** a School of Medicine, Universidad Espíritu Santo, Samborondón, 092301, Ecuador; b Instituto Nacional de Biodiversidad (INABIO), Quito, Ecuador; c Faculty of Engineering and Applied Sciences, Biotechnology, Universidad De Las Americas (UDLA), Quito, 170521, Ecuador; d One Health Research Group, Facultad de Medicina, Universidad De Las Américas (UDLA), Quito, 170124, Ecuador

**Keywords:** *Centrocestus formosanus*, *Melanoides tuberculata*, Trematode, Snails, Ecuador

## Abstract

We report the first molecularly confirmed record of the zoonotic heterophyid trematode *Centrocestus formosanus* infecting the invasive freshwater snail *Melanoides tuberculata* in tropical coastal Ecuador. A total of 902 *M*. *tuberculata* was examined, and cercarial shedding was induced under laboratory conditions. Two snails (0.22%) released cercariae morphologically consistent with Heterophyidae, and subsequent PCR amplification and sequencing of the ITS2 region confirmed *C. formosanus*. Phylogenetic analysis using the maximum-likelihood method (GTR model, 500 bootstrap replicates) revealed high genetic similarity to reference sequences from Egypt, Vietnam, Japan, and Denmark. Our findings contribute to the knowledge of expanding of this medically important zoonotic parasite in South America and identifying *M. tuberculata* as a competent intermediate host in the region. Given the role of *C. formosanus* in fish pathology and its capacity to infect humans through the consumption of raw or undercooked freshwater fish, this record raises concerns regarding potential public health and aquaculture impacts. Further studies are warranted to assess infection prevalence, identify local fish and definitive hosts, and evaluate the zoonotic risk in Ecuadorians.

## Highlights

• Trematode *C. formosanus* detected in invasive *M. tuberculata* snail from Ecuador.
• Freshwater fish consumption poses potential risk for humans.
• Molecular tools revealed zoonotic *C. formosanus* trematode in Ecuador.
• Calls for integrated One Health surveillance strategies.

## Graphical abstract

**Figure:**
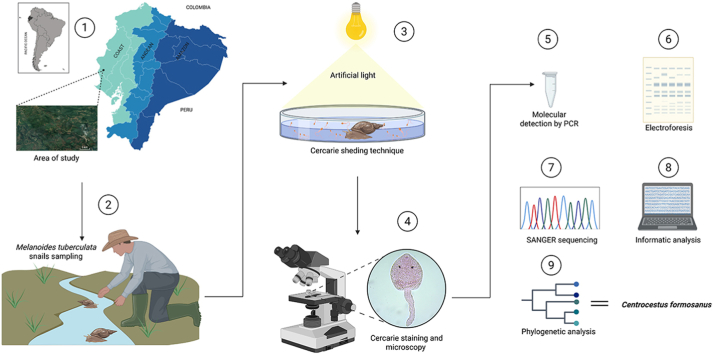

## 1 Introduction

*Centrocestus formosanus* (Nishigori, 1924) (Pinto and De Melo, 2011) is a digenean trematode, belonging to the family Heterophyidae, considered an emerging parasite of medical and veterinary importance, particularly due to its impact on aquaculture and the potential risk to human infections (Metz et al., 2022). *Centrocestus formosanus* originally described in Taiwan, has expanded its range and has been reported in Asia, Europe, in North America including USA and Mexico, and more recently into South America comprising Brazil, Venezuela, Colombia, and Peru (Metz et al., 2022; Pulido-Murillo et al., 2018). It has a complex, three-host life cycle: the first intermediate hosts are freshwater snails, particularly species of the family Thiaridae (e.g. *Melanoides tuberculata*) that are essential for the development of the larval stages (miracidia, sporocysts, and cercariae), introduced snails are potential source of novel parasites (Pulido-Murillo et al., 2018); the second intermediate hosts are various freshwater fish, where the parasite encysts in the gills as metacercariae; in Ecuador, *C. formosanus* was already reported in freshwater fish *Andinoacara rivulatus* (Romero-Alvarez et al., 2020); the definitive hosts involve piscivorous birds and mammals that become infected by consuming raw fish where the metacercariae develop into adult worms in the small intestine. The growing popularity of raw fish as items for human consumption, and the presence of *M. tuberculata* raises concern for the possibility of human cases (Pinto and De Melo, 2011). Human infections with *C. formosanus* have been reported in Asia, particularly in Laos, China, and Vietnam, with symptoms including epigastric pain, indigestion, and diarrhea (Chai et al., 2013). A critical factor in the spread of *C. formosanus* is its association with invasive freshwater snails.

The snail, *Melanoides tuberculata* (O. F. Muller, 1774) (Caenagastropoda: Thiaridae) is one of the red-rimmed melania or Malayan trumpet snail, native to Southeast Asia and Africa, has become highly invasive and is now established in many freshwater ecosystems worldwide (Vogler et al., 2012; Pinto et al., 2023). *Melanoides tuberculata* plays a crucial role as the first intermediate host in the life cycle of trematodes for at least 37 species belonging to 17 distinct families, including 11 that are known to infect humans, including *C*. *formosanus* (Pinto and De Melo, 2011; Yousif et al., 2010). Four human-infectious trematodes are transmitted by *M. tuberculata* in USA including *Stellantchasmus falcatus*, *C. formosanus*, *Haplorchis pumilio*, and *Philophthalmus gralli* (Alicata and Schatttenburg, 1938; Metz et al., 2022). In Ecuador, *M. tuberculata* has become increasingly widespread in freshwater bodies, yet the presence of *C. formosanus* has not been documented. Ecuador's unique geographic and climatic zones, from humid tropical lowlands to high-altitude paramo regions, create a mosaic of habitats that allow invasive species to thrive. *M. tuberculata* has been identified in the Pacific Coast and Amazonian regions in different freshwater habitats, including rivers, streams, lakes, reservoirs, and man-made culture ponds of the shrimp *Penaeus vannamei* and fish *Dormitator latifrons* (Correoso, 2008; Lodeiros et al., 2025).

Molecular-genetic techniques, including DNA-based methods, provide tools for accurately identifying and characterizing trematode infections, facilitating a deeper understanding of the epidemiology of these parasites. The 18S rRNA and ITS2 gene regions, are useful for identification of cercariae of some trematodes found in this thiarid (Chontananarth and Wongsawad, 2010; Tolley-Jordan et al., 2022).

This study presents the first record of *C. formosanus* infection in *M. tuberculata* collected in tropical Ecuador. Phylogenetic analysis of the parasite-DNA was conducted to confirm the species. The findings contribute to understanding the ecological and public health risks posed by this trematode-snail association in Ecuadorian freshwater systems.

## 2 Materials and methods

### 2.1 Study area and sampling

**Fig. 1:**
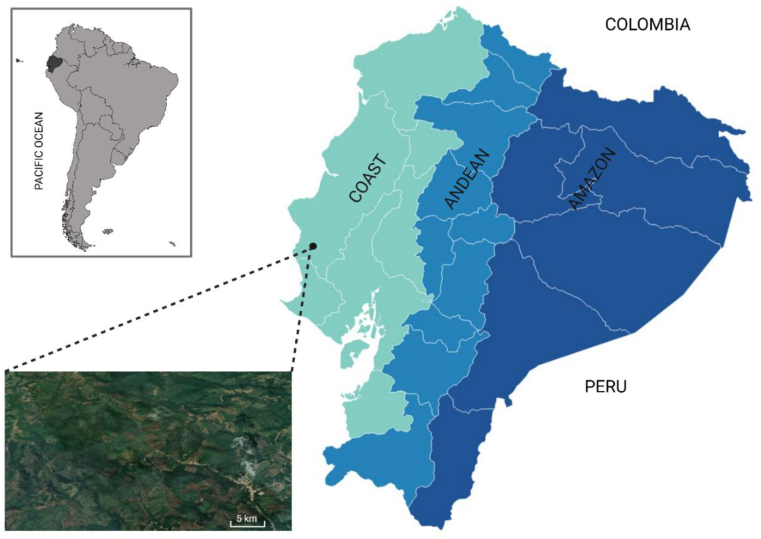
Map of Ecuador. The map illustrates the collection site (black dot) of *Melanoides tuberculata* in Pedro Pablo Gómez parish (Manabí province). Inset provides satellite imagery of the water bodies where snails were collected, with a 1000 m scale reference for spatial context.

A tropical area in the coastal region bordering the Pacific Ocean of Ecuador was selected for this study. Because it has been noted for the presence of *M*. *tuberculata* (Calvopiña et al., 2021). This area is situated within the Manabí province, Jipijapa Canton, Pedro Pablo Gomez (PPG) parish (−1.62660; −80.56018). The surveyed streams traverse rural villages that have been previously documented in our research, where endemicity for both human and animal trematodiasis (paragonimiasis, amphimeriasis, and *Haplorchis pumilio*) has been established (Calvopiña et al., 2014; 2021; Rodríguez-Hidalgo et al., 2024). The streams are part of the PPG River system, which is distinguished by low flow, with some retaining water year-round. The region exhibits a multitude of water reservoirs that provide highly favorable environmental conditions, which support optimal habitats for freshwater snails (Fig. 1).

### 2.2 Snail sampling and cercarial shedding

Freshwater snails were collected from four sites within the PPG low-stream systems during two field campaigns conducted in the dry seasons of 2023 and 2024. Collections were scheduled during the dry season due to improved site accessibility and optimal conditions for snail observation and sampling. Snails were systematically collected from both peripheral and central zones of slow-flowing or stagnant stream sections, as well as from adjacent pond habitats. Sampling was performed using a standard flat wire mesh scoop, as described by Caron et al. (2008). Snails were placed into plastic screw-capped containers and transported alive to the Laboratory of Research at Universidad de las Américas in Quito. The collection was approved by the Ecuadorian Ministry of the Environment (Contract MAE-DNB-CM-2018-0090). Snails were identified as *Melanoides tuberculata* through the application of taxonomic keys following (Pointier et al., 2003).

Cercarial shedding was conducted by placing in groups of five individuals of *M. tuberculata* for the cercarial-emission as described by Kiatsopit et al. (2014). Each group was maintained in a sterile 6-well culture plate (Thermo Scientific, USA) containing 5 mL of distilled water and fragments of lettuce. Cercariae was observed for up to five days, with the water being replaced daily. The snails were subjected to an artificial bright light source (25 W) positioned 30 cm away and exposed for 3 h per day at room temperature (12–20 °C). The presence of cercariae was then determined for each well through examination under a stereomicroscope. When cercariae were detected in a pool, the snails were subsequently separated and individually examined to identify the emitting one. Individual cercaria were separated in 10% formalin and 70% ethanol, for further analysis. Cercariae recovered were subjected to morphological identification using Lugol's iodine and Diff-Quik® reagent (Sysmex, Kobe, Japan). Stained specimens were mounted on glass slides and examined under a light microscope at 40 × and 100 × magnifications. Key anatomical structures were observed, such as the oral and ventral suckers, excretory system, and tail morphology. Images were captured for comparative analysis with existing morphological descriptions in the literature, providing preliminary identification before molecular confirmation.

### 2.3 DNA extraction and molecular identification of cercariae

The ethanol-preserved cercariae was centrifuged at 10000 rpm for 10 s. The supernatant was then removed, leaving a drop of it since the pellet was not visible. Subsequently, the ethanol was completely evaporated via incubation at 56 °C with the cap open. Lastly, the pellet was resuspended in 30 *μ*L of TE and vortexed for 5 s. Cercariae were characterized through the amplification of a 500bp fragment of the internal transcribed spacer 2 (ITS2) region using the primers ITS2-3S (5′-GGTACCGGTGGATCACTCGGCTCGTG-3′) and ITS2-A28 (5′-GGGATCCTGGTTAGTTTCTTTTCCTCCGC-3′) (Sugiyama et al., 2005). The product was amplified in 15 *μ*L reactions of 15 *μ*L with the high-fidelity Phusion Taq polymerase (Invitrogen, USA) and 0.3 *μ*M of each primer. The thermocycling conditions were as follows: an initial denaturation at 98 °C for 5 min, 33 cycles of denaturation at 98 °C for 30s, annealing at 51 °C for 30s, and extension at 72 °C for 30s, with a 7 min extension at 72 °C.

### 2.4 Sequencing and phylogenetic analysis

The PCR products were sequenced using the Sanger technique on an ABI 3500xL Genetic Analyzer (Applied Biosystems, Foster City, CA, USA) with BigDye 3.1® capillary electrophoresis matrix. The sequences were edited using Molecular Evolutionary Genetics Analysis version 11 (MEGA 11) software (Tamura et al., 2021) and compared against the GenBank database at the National Center for Biotechnology Information (NCBI) via the Basic Local Alignment Search Tool (BLAST). Subsequently, the sequences were aligned using the Multiple Alignment using Fast Fourier Transform (MAFFT) algorithm, and the optimal model was chosen based on the Akaike Information Criterion (AIC). Tree topologies and branch lengths were computed using the maximum-likelihood method (ML) with 500 bootstrap replicates. The model employed was General Time Reversible (GTR) with a gamma distribution (G). The phylogenetic tree was generated using the NGPhylogeny online software (Lemoine et al., 2019) and modified on the iTOL software (Letunic and Bork, 2021).

## 3 Results

**Fig. 2:**
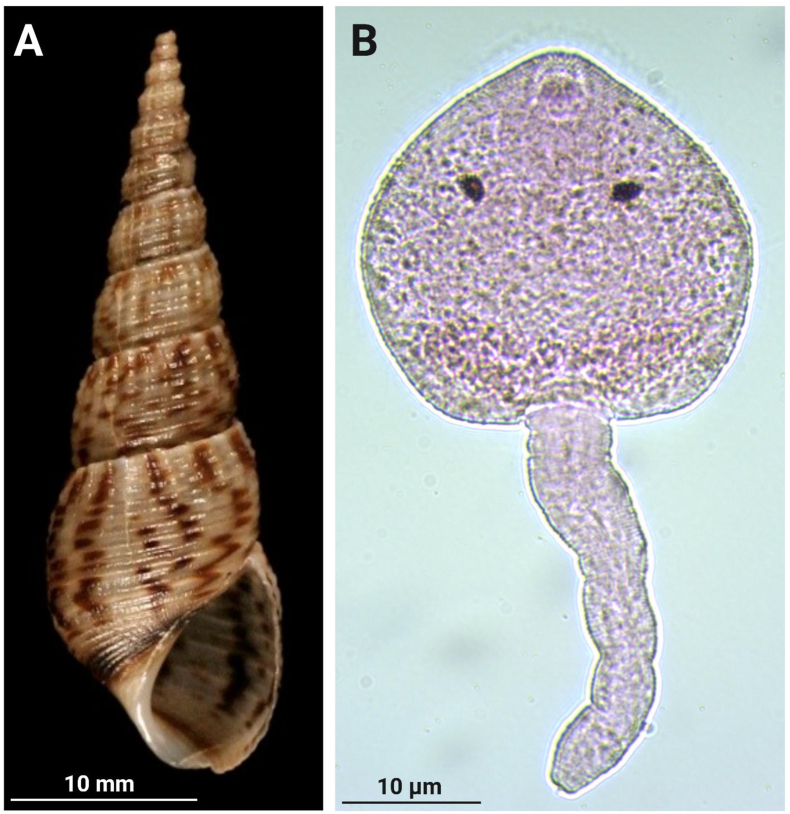
A. *Melanoides tuberculata* snail. The shells are pale brown with numerous reddish-brown flames and spots; the body whorls are well rounded and ornamented with spiral grooves and have axial and spiral rows of small tubercles; there is also a brown band in the columellar region. B. Photomicrograph of a cercaria stained with Diff-Quik (100x). The larva displays a large ovoid body and a non-bifurcated tail with fine striations. Prominent pigmented eyespots are evident. The ventral sucker is clearly visible in the anterior region located posterior to the oral sucker, morphological features consistent with members of the family Heterophyidae.

A total of 902 snails were collected from the study sites identified later as *M. tuberculata* (Fig. 2A). Following the cercarial shedding process, two snails emitted cercariae. Cercarial release was observed on the third and fifth day of exposure. Morphological analysis revealed the presence of a single cercarial morphology characteristic of a trematode (Fig. 2B).

Subsequent molecular analysis based on PCR amplification and sequencing of a ∼500 bp fragment of the ITS2 region confirmed that the recovered cercariae belonged to *Centrocestus formosanus*. Sequence alignment was performed with the MAFFT algorithm, and the optimal evolutionary model was selected according to the Akaike Information Criterion (AIC). The General Time Reversible (GTR) model with a gamma distribution (G) was applied. The obtained sequence was deposited in GenBank under accession number PQ383505 (*Centrocestus formosanus* isolate UDLA1, 5.8S ribosomal RNA gene and internal transcribed spacer 2, partial sequence).

**Fig. 3:**
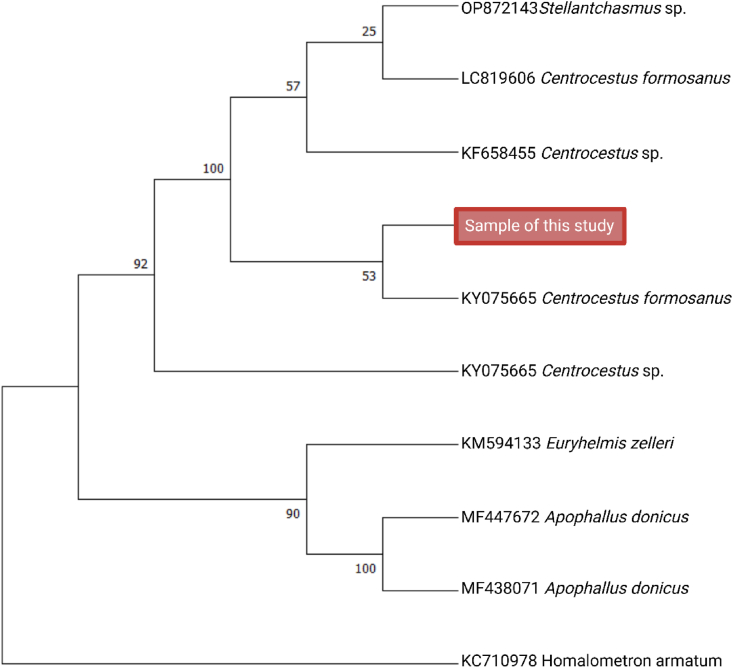
Phylogenetic tree based on ITS2 sequences constructed using 500 bootstrap replicates. The Ecuadorian sample (GenBank accession number PQ383505) show a great similarity with sequences of *C. formosanus* from Egypt, Vietnam, Japan, and Denmark.

Phylogenetic analysis further supported this identification, revealing a strong similarity with reference sequences of *C. formosanus* from Egypt (LC819606), Vietnam (KY075665), and a *Centrocestus* sp. from Japan (LC805536), as well as with a sequence described in Denmark (KF658455) derived from imported fish. The recovered sequence clustered within a well-supported clade containing these isolates, with high bootstrap values (Fig. 3). Additionally, pairwise genetic distance analysis revealed minimal divergence among all sequences included in the alignment (≈0.0–0.2%). The complete pairwise comparison matrix is provided as Supplementary Table 1.

## 4 Discussion and conclusions

This study provides the first molecular-genetic confirmation of *Centrocestus formosanus* infecting *Melanoides tuberculata* snails collected from a tropical Coastal ecoregion of Ecuador; thus, expanding the known geographical distribution of this zoonotic intestinal trematode in South America. No human cases have been diagnosed in Ecuador; however, the widespread consumption of freshwater fish, especially in raw or undercooked preparations (Calvopiña et al., 2011; Rodríguez-Hidalgo et al., 2024) raises concerns about its zoonotic potential. In previous studies conducted in the same geographic region, infection by *C. formosanus* has been reported in the freshwater fish *Andinoacara rivulatus* (Romero-Alvarez et al., 2020). This finding provides additional evidence that components of the parasite's life cycle may already be established locally.

Human infections have been reported in Laos, China, and Vietnam (Chai et al., 2013). In USA four human trematodiasis including *C. formosanus* are transmitted by *M. tuberculata* (Alicata and Schatttenburg, 1938; Metz et al., 2022). Previous reports have documented this parasite in various countries of the Americas, comprising Brazil, Colombia, Costa Rica, and USA (Arguedas Cortés et al., 2010; Metz et al., 2022; Pinto and De Melo, 2011; Vergara and Velásquez, 2009). Detecting *C. formosanus* in the invasive *M. tuberculata* evidenced further dispersal and a potential risk infection for fish, birds, humans, and other mammals. For example, in Costa Rica, high metacercarial loads were documented in tilapia fry, leading to significant fish mortality (Arguedas Cortés et al., 2010). In Ecuador, a similar situation was observed with *Amphimerus* sp., a liver fluke initially overlooked as a public health concern in Ecuador, but later confirmed to be endemic trematodiasis infecting fish, humans, dogs, and cats (Calvopiña et al., 2015; Rodríguez-Hidalgo et al., 2024; Romero-Alvarez et al., 2020). Given that cercariae of *C. formosanus* infected *M. tuberculata*, the potential for transmission to fish and ultimately to humans warrants further investigation. This concern is heightened by recent reports documenting the presence of *M. tuberculata* in shrimp and fish aquaculture ponds (Lodeiros et al., 2025). *M. tuberculata* in aquaculture environments may facilitate the maintenance of the parasite's life cycle, posing potential risks for fish health, aquaculture productivity, and the transmission of *C. formosanus* and other trematode through the consumption of infected freshwater fish.

From a public health perspective, *C. formosanus* is a recognized zoonotic parasite capable of infecting humans through the consumption of raw or undercooked freshwater fish harboring its metacercariae. Human infections have been reported in Asia where intestinal heterophyid infections are associated with gastrointestinal symptoms and, in heavy infections, more severe clinical outcomes (Chai et al., 2013). The public health relevance of these findings is further underscored by parallels with other trematode infections in Ecuador (Calvopiña et al., 2015, 2021; Rodríguez-Hidalgo et al., 2024). These precedents suggest that intestinal trematode human infections may be underdiagnosed in Ecuador, particularly in rural and coastal areas where access to diagnostic tools is limited. Consequently, the detection of *C. formosanus* cercariae in *M. tuberculata* snails should be regarded as an early warning of potential zoonotic transmission.

The widespread distribution of the invasive snail *M. tuberculata* in Ecuadorian freshwater bodies (Correoso, 2008) creates favorable conditions for the establishment and persistence of *C. formosanus* and other trematodes using this species as an intermediate host (Metz et al., 2022; Yousif et al., 2010). This snail has been reported in both the Pacific coastal and Amazonian regions of Ecuador, occupying diverse freshwater habitats (Correoso, 2008). Moreover, climate change may further enhance the risk of transmission by facilitating snail expansion into higher altitudes and previously unsuitable environments, thereby increasing opportunities for parasite dispersal and host–parasite contact (Mas-Coma et al., 2001; Poulin, 2006).

The low prevalence observed in this study (0.22%; 2/902) contrasts with higher infection rates reported elsewhere, such as in Brazil, where prevalence reached approximately 7% (Pinto and De Melo, 2011), it may represent a nascent phase of introduction of *C*. *formosanus* in populations of *M*. *tuberculata*, rather than a stable endemic situation. Variations in infection rates may reflect differences in the abundance and availability of suitable definitive hosts, local environmental conditions, parasite population structure, and ecological factors influencing transmission. Anthropogenic impacts, including water pollution, agricultural runoff, habitat modification, and aquaculture practices, may further shape transmission dynamics and deserve further investigation in Ecuadorian freshwater systems (Yang et al., 2012; Torremorell et al., 2021).

Ecuadorian isolates of *C. formosanus* revealed a high degree of genetic similarity to those reported from Asia, Europe, Africa, and North America, supporting the hypothesis of a globally distributed but underreported parasite. This is consistent with the pairwise genetic distance analysis, which showed minimal divergence among sequences (≈0.0–0.2%), confirming a high level of genetic homogeneity across geographically distant populations. Previous studies have demonstrated minimal intraspecific genetic divergence among *C. formosanus* populations, with isolates from Laos showing 100% sequence homology with those from the United States (Chai et al., 2013).

Additionally, the detection of *C. formosanus* in ornamental fish imported into Europe highlights the role of international trade and aquaculture in the dissemination of this parasite (Pace et al., 2020). The close phylogenetic relationship observed in this study suggests that similar pathways may have contributed to its introduction into Ecuador, emphasizing the importance of global connectivity in shaping parasite distribution patterns. While the ITS2 marker provided reliable species-level identification, future studies should incorporate additional molecular markers, such as mitochondrial COI, to improve phylogenetic resolution and to assess potential intraspecific genetic variation and introduction pathways of *C. formosanus* populations in Ecuador.

In conclusion, this report constitutes the first confirmed record of *C. formosanus* in Ecuador and provides baseline evidence of its establishment within local freshwater ecosystems. Further research is needed to evaluate infection prevalence and seasonal dynamics in *M. tuberculata* populations, to identify metacercarial infections in edible freshwater fish species, and to determine the definitive hosts involved in local transmission cycles. Given the zoonotic potential of *C. formosanus* and the possible influence of climate change on host–parasite interactions, integrated surveillance combining parasitological, ecological, and public health approaches is warranted. Such efforts will be essential to assess potential risks to human health, fisheries, and aquaculture, and to inform future prevention and control strategies in Ecuador.

## CRediT authorship contribution statement

**Carlos Bastidas-Caldes:** Writing – review & editing, Visualization, Validation, Resources, Methodology, Investigation, Funding acquisition, Conceptualization. **Fernanda Hernández-Alomia:** Writing – review & editing, Methodology, Investigation, Data curation. **Juan Pablo Negrete:** Writing – review & editing, Methodology, Investigation, Data curation. **Manuel Calvopina:** Writing – review & editing, Writing – original draft, Validation, Supervision, Resources, Project administration, Investigation, Funding acquisition, Data curation, Conceptualization.

## Financial support

Funded by the Universidad de las Americas (UDLA); grant MED.MCH.23.01 (MC).

## Declaration of competing interest

All authors declare no known competing financial interests or personal relationships that could have appeared to influence the work reported in this paper.

## Data Availability

Data are available on request.
